# Anti-Cancer Nanopowders and MAPLE-Fabricated Thin Films Based on SPIONs Surface Modified with Paclitaxel Loaded β-Cyclodextrin

**DOI:** 10.3390/pharmaceutics13091356

**Published:** 2021-08-28

**Authors:** Rebecca Alexandra Puiu, Paul Cătălin Balaure, Ema Constantinescu, Alexandru Mihai Grumezescu, Ecaterina Andronescu, Ovidiu-Cristian Oprea, Bogdan Stefan Vasile, Valentina Grumezescu, Irina Negut, Ionela Cristina Nica, Miruna Silvia Stan

**Affiliations:** 1 Department of Science and Engineering of Oxide Materials and Nanomaterials, Faculty of Applied Chemistry and Materials Science, Politehnica University of Bucharest, 011061 Bucharest, Romania; rebecca_alexandra92@yahoo.com (R.A.P.); constantinescu.ema94@gmail.com (E.C.); a.grumezescu@upb.ro (A.M.G.); ecaterina.andronescu@upb.ro (E.A.); bogdan.vasile@upb.ro (B.S.V.); 2 “Costin Nenitzescu” Department of Organic Chemistry, Faculty of Applied Chemistry and Materials Science, Politehnica University of Bucharest, 011061 Bucharest, Romania; 3 Research Institute of the University of Bucharest—ICUB, University of Bucharest, 050657 Bucharest, Romania; cristina.nica@drd.unibuc.ro (I.C.N.); miruna.stan@bio.unibuc.ro (M.S.S.); 4 Academy of Romanian Scientists, Ilfov No. 3, 50044 Bucharest, Romania; 5 Department of Inorganic Chemistry, Physical Chemistry and Electrochemistry, Politehnica University of Bucharest, 011061 Bucharest, Romania; ovidiu.oprea@upb.ro; 6 Lasers Department, National Institute for Lasers, Plasma and Radiation Physics, 077125 Magurele, Romania; valentina.grumezescu@inflpr.ro (V.G.); negut.irina@inflpr.ro (I.N.); 7 Department of Biochemistry and Molecular Biology, Faculty of Biology, University of Bucharest, 050095 Bucharest, Romania

**Keywords:** paclitaxel, β-Cyclodextrin, SPIONs

## Abstract

Globally, cancer is the second most common cause of death, and Europe accounts for almost 25% of the global cancer burden, although its people make up only 10% of the world’s population. Conventional systemically administered anti-cancer drugs come with important drawbacks such as inefficiency due to poor bioavailability and improper biodistribution, severe side effects associated with low therapeutic indices, and the development of multidrug resistance. Therefore, smart nano-engineered targeted drug-delivery systems with tailored pharmacokinetics and biodistribution which can selectively deliver anti-cancer agents directly to the tumor site are the solution to most difficulties encountered with conventional therapeutic tools. Here, we report on the synthesis, physicochemical characterization, and in vitro evaluation of biocompatibility and anti-tumor activity of novel magnetically targetable SPIONs based on magnetite (Fe_3_O_4_) nanoparticles’ surface modified with β-cyclodextrin (CD) and paclitaxel (PTX)–guest–host inclusion complexes (Fe_3_O_4_@β-CD/PTX). Both pristine Fe_3_O_4_@β-CD nanopowders and PTX-loaded thin films fabricated by MAPLE technique were investigated. Pristine Fe_3_O_4_@β-CD and Fe_3_O_4_@β-CD/PTX thin films were physicochemically characterized by X-ray diffraction (XRD), Fourier-transform infrared spectroscopy (FT-IR), thermal analysis, scanning electron microscopy (SEM), and transmission electron microscopy (TEM). The biocompatibility of bare magnetic nanocomposite thin films was evaluated by MTT cell viability assay on a normal 3T3 osteoblast cell line culture and by measuring the level of NO in the culture medium. No significant modifications, neither in cell viability nor in NO level, could be observed, thereby demonstrating the excellent biocompatibility of the SPIONs thin films. Inverted phase-contrast microscopy showed no evident adverse effect on the morphology of normal osteoblasts. On the other hand, Fe_3_O_4_@β-CD/PTX films decreased the cell viability of the MG-63 osteosarcoma cell line by 85%, demonstrating excellent anti-tumor activity. The obtained results recommend these magnetic hybrid films as promising candidates for future delivery, and hyperthermia applications in cancer treatment.

## 1. Introduction

In a recent press release [1,2], the International Agency for Research on Cancer and the World Health Organization put cancer as the second leading cause of death globally, with an estimated 9.6 million deaths in 2018, while the global cancer burden has risen 18.1 million new cases. Most of the new cases (23.4%) and deaths (20.3%) by cancer were registered in Europe. There is a strong need for more efficient, innovative therapeutic tools to optimize clinical outcomes, increase patient compliance, and improve patient quality of life in the context of this crisis.

Most conventional medicines currently used in cancer treatment suffer some major drawbacks such as poor bioavailability associated with low water solubility, improper biodistribution, and lack of controlled, selective delivery to targeted disease areas resulting in a low therapeutic index, severe adverse effects, the development of multidrug resistance (MDR), and even treatment failure. Pharmaceutical nanotechnology provides powerful tools for circumventing these drawbacks by increasing drug bioavailability, prolonging drug circulation time, decreasing the clearance rate, and increasing drug stability in physiological media. Nanocarriers’ surfaces can be modified with targeting ligands which are guided by specific ligand–receptor interactions, allowing precise spatial control of nanocarrier localization within the body [3]. Moreover, smart nano-transporters can be engineered to trigger drug release in response to small microenvironmental changes in local pH, temperature, redox potential, and enzyme activity, as well as to remote, externally applied stimuli such as electric fields, laser pulses, ultrasounds, and magnetic fields [3,4,5]. Furthermore, nanovehicles can overcome MDR mechanisms, including decreased uptake and increased efflux of tumor cell drugs [6].

Among the various types of nanocarriers (organic polymers, inorganic polymers, lipids, metals, metal oxides), superparamagnetic iron oxide nanoparticles (SPIONs) have attracted increasing attention in recent years due to the benefits of site-specific magnetic guidance and drug release [7]. SPIONs display a number of remarkable characteristics such as biocompatibility [8] and sensitivity to an applied magnetic field [9] which makes them ideal candidates for theragnostic applications, meaning that they can be simultaneously used for imaging, targeting, and drug delivery [10,11,12,13]. Appropriately functionalized SPIONs have received FDA approval for clinical application as contrast agents in magnetic resonance imaging [14]. SPIONs can be passively targeted to tumoral zones due to their enhanced permeability and retention effect. After local accumulation, magnetic hyperthermia consisting of sudden temperature increase in the targeted diseased area exposed to an alternating magnetic field can induce the death of tumoral cells [15]. However, simple magnetite (Fe_3_O_4_) nanoparticles are unstable in polar (aqueous) physiological fluids since they tend to reduce surface energy. They are intrinsically prone to aggregation through van der Waal’s and magnetic dipole–dipole interactions [16,17,18]. Therefore, SPIONs surfaces should be modified to prevent agglomeration by covering them with a stabilizing shell of appropriate, biocompatible materials.

β-Cyclodextrin (β-CD) is a macrocyclic oligosaccharide formed of seven D-glucopyranose units linked to each other by α-1,4 glycosidic bonds [19]. It has the shape of a hollow truncated cone with a hydrophilic outer surface and a hydrophobic inner cavity. The hydrophobic interior of the cavity is lined with C-H groups with glycosidic oxygen in between, while the hydrophilic exterior is made up of the hydroxyl groups of the glucopyranose rings. All secondary hydroxyl groups are situated on the wider rim of the hollow truncated cone, whereas all primary hydroxyl groups are situated on the narrower rim. Due to these peculiar structural and shape features, β-CD molecules have the unique ability to entrap hydrophobic compounds in their internal cavity and solubilize them in an aqueous environment.

The formation of host–guest inclusion complexes relies on the complementarity of the two partners involved in the steric match. That is, the inclusion guest molecule must have a size that fits, at least partially, into the host cavity and interactionally matches the developing intermolecular forces during the inclusion process. Moreover, the inclusion complex’s binding energy, which crucially affects the loading and release performances, can be predicted by molecular docking studies [20,21]. Furthermore, cyclodextrins possess excellent biocompatibility, being largely studied as water-dispersible nanocarriers for the delivery of hydrophobic compounds and drugs like curcumin [22], paclitaxel [23], irinotecan [24], and ibuprofen [25]. An interesting study [26] uses the molecular docking and release of the guest molecule to construct pH-responsive CD-capped mesoporous silica nanoparticles.

All the above properties of β-CD make it an excellent surface modifier for SPIONs. It allows magnetite nanoparticles to become water-dispersible magnetically targetable nanovehicles suitable for hydrophobic anti-cancer drug delivery, magnetic hyperthermia, and theragnostic applications [9,10,27,28,29,30,31]. Some studies report that guest molecular docking also induces the self-assembly of SPIONs [22,23,32].

Here, we report the synthesis, characterization, biocompatibility, and in vitro anti-cancer outcome of magnetic nanocomposite vehicles based on PTX/β-CD inclusion complexes physically adsorbed onto Fe_3_O_4_ nanoparticles. We studied both nanopowder composites and thin films deposited by matrix-assisted pulsed laser evaporation (MAPLE) technique [33].

## 2. Materials and Methods

### 2.1. Materials

All chemicals used for the synthesis of the nanostructured materials were purchased from Sigma-Aldrich (Merck Group, Darmstadt, Germany), namely anhydrous ferric chloride (FeCl_3_, >99.99% trace metal basis), ferrous sulfate heptahydrate (FeSO_4_·7H_2_O, >98%), ammonium hydroxide solution (25% NH_3_ in H_2_O), β-cyclodextrin, paclitaxel, dimethyl sulfoxide (DMSO), chloroform (CHCl_3_), analytical graded acetone (C_6_H_6_O), and ethanol (C_2_H_6_O).

### 2.2. Chemical Synthesis of β-Cyclodextrin-Covered Fe_3_O_4_ Nanoparticles

Fe_3_O_4_–β-cyclodextrin nanoparticles (Fe_3_O_4_@β-CD) were prepared by the co-precipitation method, which involves base-induced simultaneous precipitation of ferrous (Fe^2+^) and ferric (Fe^3+^) ions in an aqueous solution [34]. In brief, solution 1 (Sn1) was prepared by dissolving ferrous sulfate and ferric chloride (molar ratio 1:2) in 300 mL demineralized water. Solution 2 (Sn2) consisted of 1 g β-CD and 9 mL ammonium hydroxide in 300 mL demineralized water. Next, Sn 1 was added dropwise into Sn2 using a pressure-equalizing dropping funnel under magnetic stirring. The precipitated magnetite nanoparticles were magnetically collected. After decantation of the liquid phase, the resultant powder was thoroughly washed three times with demineralized water and left to air dry at room temperature. The nanopowders were fully characterized by X-ray powder diffraction (XRD), scanning electron microscopy (SEM), transmission electron microscopy (TEM), Fourier transform infrared spectroscopy (FT-IR), and thermal analysis (simultaneous thermogravimetric analysis (TGA) and dynamic scanning calorimetry (DSC).

### 2.3. Preparation of Paclitaxel Inclusion Complex

PTX-β-CD inclusion complexes were prepared by the solvent evaporation method. To this end, 100 mg Fe_3_O_4_@β-CD nanoparticles were redispersed in a solution of PTX (10 mg, selected based on previous research results reported by [35]) in chloroform (1 mL) was added and mixed in a grinding mortar until complete evaporation of chloroform. This was subsequently stored at 5 °C for further use.

### 2.4. Target Preparation and Deposition of Fe_3_O_4_@ Paclitaxel-Loaded β-CD

Compared to solvent-based methods used to prepare thin films such as spin-coating, dip-coating, and drop-casting, MAPLE technique has the advantage of homogeneity on the deposited area and the possibility of tailoring the final thickness, which is of crucial importance for the efficacy and mechanical resistance of bioactive coatings [36].

We deposited Fe_3_O_4_@PTX-loaded β-CD thin films on glass and Si substrates which previously have been successively cleaned with acetone, ethanol, and deionized water in an ultrasonic bath for 15 min. The substrates were dried under a high purity nitrogen stream. The MAPLE targets consisted of a 2% suspension of Fe_3_O_4_@β-CD/PTX inclusion complex nanocomposites in DMSO which was poured into a pre-cooled target holder at 173 K and subsequently immersed in liquid nitrogen for 30 min. The MAPLE depositions were performed using a KrF* excimer (λ = 248 nm and τ_FWHM_ = 25 ns) COMPexPro 205 model, Lambda Physics-Coherent (Göttingen, Germany), that was operated at a repetition rate of 15 Hz. Three different fluences of the laser beam, i.e., 300, 400, and 500 mJ/cm^2^, respectively, were investigated. A number of laser pulses ranging between 22,000 and 75,000 were applied to each target. During MAPLE processing, the laser spot area was 30 mm^2^ and the target holder was rotated with a frequency of 0.4 Hz. During the deposition process, the experimental parameters (i.e., substrate temperature, background pressure, and target to substrate distance) were maintained constant (room temperature, 1 Pa, 4 cm, respectively).

### 2.5. Physicochemical Characterization

The crystallinity of Fe_3_O_4_@β-CD nanopowders was investigated by XRD analysis carried out on a Shimadzu XRD 6000 diffractometer. X-ray diffraction patterns were recorded at room temperature using CuKα radiation (λ = 1.54056Å at 15 mA and 30 kV) with the Bragg diffraction angle 2θ ranging between 10 and 80°.

The inner structure and morphology of the nanocomposites were investigated by TEM. The nanopowder TEM specimens were prepared by dispersal in ethanol and ultrasonic cleansing for 15 min. Next, the sample was placed on a carbon-coated copper grid and left to dry at room temperature. TEM images were obtained using a TecnaiTM G2 F30 S-TWIN electron microscope equipped with selected area electron diffraction (SAED) purchased from the FEI (Hillsboro, OR, USA) company. The microscope was operated in transmission mode at 300 kV. The guaranteed TEM point resolution and TEM line resolution were 2 Å and 1.02 Å, respectively.

The shape and size nano-details of the surface of the Fe_3_O_4_@β-CD composites were evaluated by SEM analyses performed on an FEI electron microscope, using secondary electron beams with energies of 30 keV.

To confirm the structural integrity of the thin films of bare and PTX-loaded β-CD surface-modified Fe_3_O_4_ nanoparticles deposited by MAPLE technique at various fluences, we monitored the surface distribution of the chemical functional groups for CD and PTX by FT-IR chemical maps. IR mapping (IRM) was performed on a Nicolet iN10 MX FT-IR microscope with an MCT liquid nitrogen cooled detector in the 4000–1000 cm^−1^ range. The spectral collection was made in reflection mode at 4 cm^−1^ resolution. A total of 32 scans were co-added and converted to absorbance for each spectrum using OmincPicta software (Thermo Scientific, Waltham, MA, USA). About 250 spectra were analyzed for each sample. The absorption peaks of C-H and C-O-C functional groups in CD and C=O carbonyl groups in PTX were chosen as specific spectral markers.

The thermal analyses were assessed with a Shimadzu DTG-TA-50H equipment (Carlsbad, CA, USA) from room temperature to 800 °C at a heating rate of 10 K min^−1^, under a flow of 20 mL min^−1^ dried synthetic air (80% N_2_ and 20% O_2_).

### 2.6. Biological Evaluation

The biocompatibility of Fe_3_O_4_@β-CD nanoparticle thin films on glass slides was evaluated in the presence of MC3T3-E1 murine osteoblast cultures. The anti-tumor efficiency of Fe_3_O_4_@ PTX-loaded β-CD thin films on glass substrates was tested on MG-63 osteosarcoma cells. Both types of cell lines were cultured in Dulbecco’s Modified Eagle Medium (DMEM) supplemented with 10% fetal bovine serum and antibiotic mixture at 37 °C in a humidified atmosphere with 5% CO_2_. All specimens, including the uncoated substrates (control), were sterilized by UV exposure for one hour before cell seeding.

The percentage of viable cells was measured with an MTT test, which involves the enzymatic reduction of the tetrazolium salt to insoluble formazan inside the metabolically active cells. The cells were seeded on top of uncoated and coated substrates at a cellular density of 4 × 10^4^ × cells/cm^2^. Images on an inverted phase-contrast microscope (Olympus IX71, Tokyo, Japan) were taken after 24 h of incubation. Subsequently, the culture medium was removed and replaced with MTT solution (1 mg/mL concentration), followed by 2 h of standard incubation in dark conditions. The water-insoluble formazan crystals were dissolved with isopropanol. The absorbance (directly related to the number of metabolically active cells) was measured at 595 nm using a FlexStation 3 multi-mode microplate reader from Molecular Devices (San Jose, CA, USA).

The concentration of nitric oxide (NO) in the collected culture medium after 24 h of incubation was performed with Griess reagent, which is a stoichiometric solution (*v/v*) of 0.1% naphthylethylenediamine dihydrochloride and 1% sulphanilamide in 5% H_3_PO_4_. Increased NO levels are related to cytotoxic effects as this molecule is connected with inflammation and apoptosis processes. The absorbance of the mix was measured at 550 nm using the FlexStation 3 multi-mode GENios microplate reader, and the NO concentration was calculated from the standard NaNO_2_ curve.

Biological test results were analyzed using Student’s *t*-test on Excel (Microsoft Office 2018). Statistically significant data were considered as having *p*-value of less than 0.05.

### 2.7. Preparation of Simulated Body Fluid and Procedure of Apatite-Forming Abilities

Simulated body fluid (SBF) was prepared according to the instructions presented by Kokubo et al. [37].

The thin films were immersed in SBF using 6-well plates. The 6-well plates were placed for 14 days in an unstirred thermostatic water bath at 37 °C. The experiment was done in triplicate. After 14 days, the samples were dried at room temperature and characterized using a Versa 3D DualBeam (FIB/SEM) scanning electron microscope.

## 3. Results

### 3.1. Physicochemical Characterization of Fe_3_O_4_@β-CD Nanopowders

**Figure 1:**
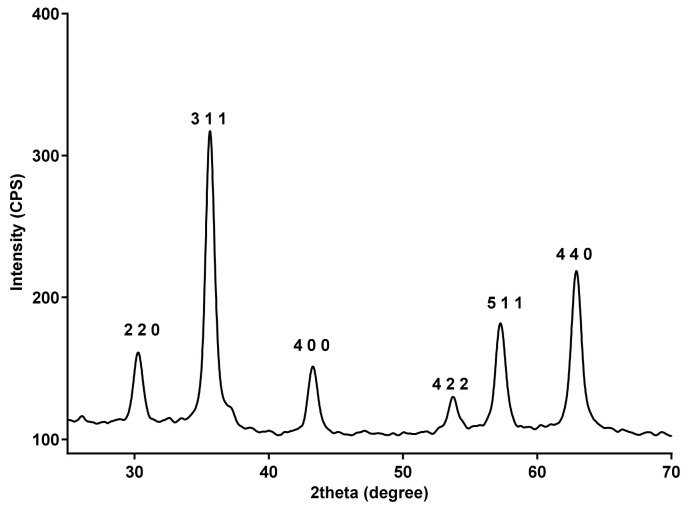
XRD patterns of fabricated Fe_3_O_4_@β-CD nanopowders.

The XRD patterns of Fe_3_O_4_@β-CD nanopowders are plotted in Figure 1. Sharp diffraction peaks appearing at 2θ = 30.31, 35.71, 43.31, 53.90, 57.61, and 62.81 were assigned to the (2 2 0), (3 1 1), (4 0 0), (4 2 2), (5 1 1), and (440) planes of the magnetite lattice, respectively, being in good agreement with the literature [38]. No other diffraction peaks corresponding to another iron oxide, such as α-Fe_2_O_3_ or γ-Fe_2_O_3_, could be observed.

**Figure 2:**
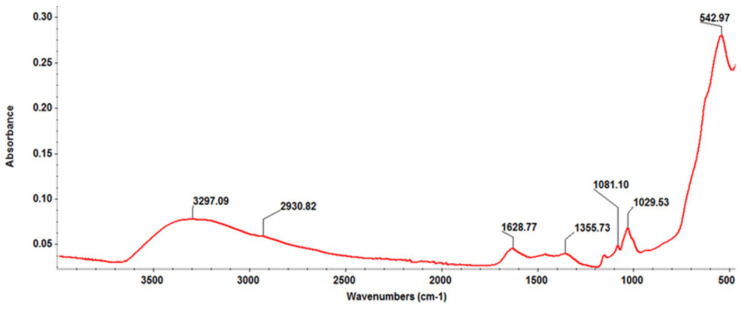
FT-IR spectrum of Fe_3_O_4_@β-CD nanopowders.

The main absorptions present in the FT-IR spectrum of Fe_3_O_4_@β-CD nanocomposites (Figure 2) were as follows: 3297 cm^−1^ stretching vibrations of cyclodextrin OH groups, 2930 cm^−1^ anisomerous stretching vibrations of aliphatic C-H and CH_2_ bonds in β-CD, 1081 cm^−1^ and 1029 cm^−1^ stretching vibrations of C-C, C-O-C bonds, and the wagging vibration of the O-H bonds directly at the sugar ring, while the intense peak at 543 cm^−1^ corresponds to the Fe-O stretching mode of the tetrahedral and octahedral sites [39,40,41,42].

**Figure 3:**
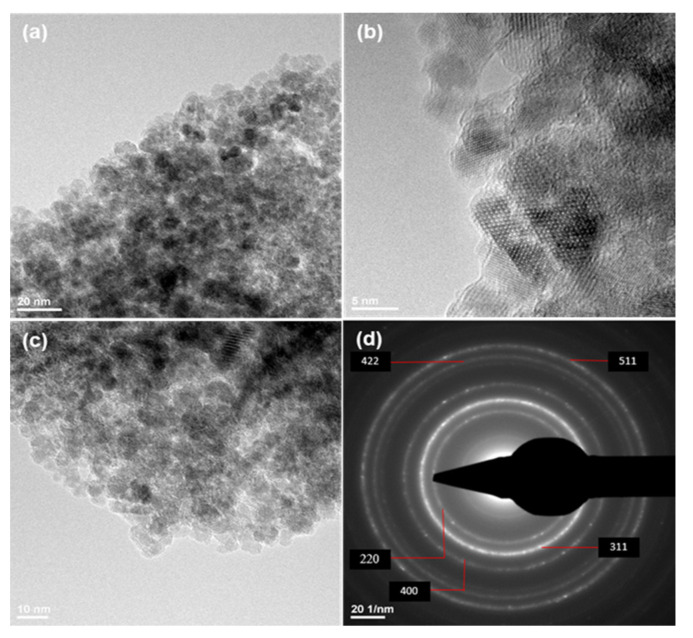
TEM (**a**–**c**) micrographs and SAED pattern (**d**) of Fe_3_O_4_@β-CD nanopowders.

The TEM images of the Fe_3_O_4_@β-CD nanopowders are plotted in Figure 3. At higher magnifications, one can distinguish a nanocrystalline magnetite phase without agglomeration and a low-ordered non-crystalline shell that can be attributed to the β-CD coating (Figure 3b). The recorded SAED ring pattern plotted in Figure 3d corresponds to the (220), (311), (400), (422), and (511) magnetite lattice planes being in perfect agreement with the high polycrystallinity of the magnetite phase and confirming once again the absence of any other crystalline phase.

**Figure 4:**
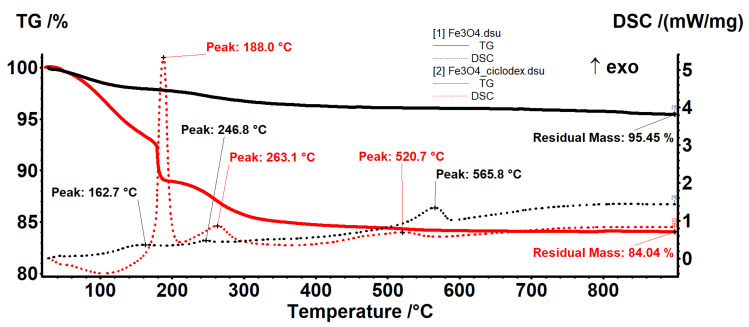
Thermal analysis for Fe_3_O_4_ and Fe_3_O_4_@β-CD nanopowder.

The simultaneous enthalpy and mass changes vs. temperature curves (DSC-TGA curves) for simple Fe_3_O_4_ and β-CD-surface-modified magnetite are plotted in Figure 4. Figure 4 shows that the first mass loss of 1.74% occurs between room temperature and 120°. It was attributed to the dehydration of Fe_3_O_4_ and loss of the hydroxyl groups on the surface of Fe_3_O_4_ nanoparticles in a slightly endothermic process. Next, two successive mass losses of 0.54% and 0.98% occur in the temperature ranges 120–200 °C and 200–300 °C, respectively, corresponding to the exothermic peaks at 162.7 °C and 246.8 °C. These peaks can be attributed to the thermal decomposition and oxidation of some organic matter impurities present on magnetite’s surface. The strong exothermic peak observed at 565.8 °C corresponds to a transformation of the Fe_3_O_4_ into maghemite (γ-Fe_2_O_3_). Unlike Fe_3_O_4_, the crystal lattice of γ-Fe_2_O_3_ presents vacant cation sites, commonly in octahedral sites. The mass loss of the latter transformation is 1.28%. Thus, the total mass loss is 4.54%, corresponding to a residual mass of 95.45% wt. of the initial mass. Fe_3_O_4_@β-CD nanocomposite’s DSC curve shows one endothermic peak at 106 °C followed by three exothermic peaks at 188, 263.1, and 520.7 °C, respectively. The sharp exothermic peak at 188 °C is due to the degradation of the β-CD organic phase deposited on the inorganic Fe_3_O_4_ phase’s surface. The conversion of magnetite to maghemite is marked by the exothermic peak at 520.7 °C. The residual mass is 84.04% wt. of the initial mass. The difference between the residual mass of pristine Fe_3_O_4_ and the residual mass of Fe_3_O_4_@β-CD nanocomposite is a rough estimate of the β-CD coating weight (11.51 g/100 g Fe_3_O_4_).

### 3.2. Physicochemical Characterization of Fe_3_O_4_@β-CD Thin Films

To evaluate the structural-functional groups’ integrity after laser processing for the thin films deposited by the MAPLE technique, compared with drop-cast, we used the FT-IR imaging technique. Absorbance maps were created based on the minima of the second derivative of the spectral data. As specific spectral markers for β-CD, we used the absorptions’ intensities at 2930 and 1029 cm^−1^, while for PTX, we choose as characteristic intensity the stretching ester/ketonic carbonyl vibration at 1739 cm^−1^ [43,44].

**Figure 5:**
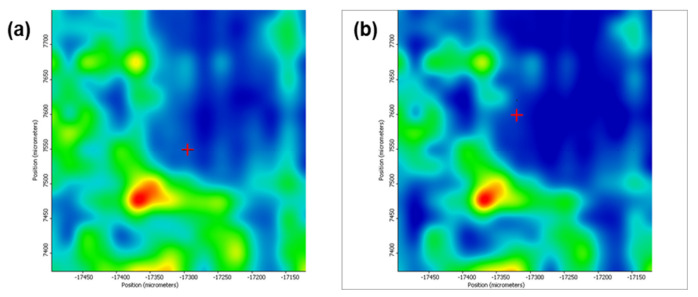
Second derivate IR mappings of Fe_3_O_4_@β-CD drop-cast: surface intensity distribution of (**a**) C-H and CH_2_ stretching vibrations at 2930 cm^−1^ and (**b**) C-O bond stretching vibrations at 1029 cm^−1^.

**Figure 6:**
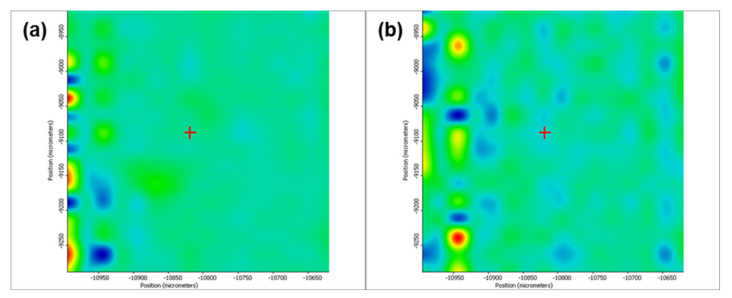
Second derivate IR mappings of Fe_3_O_4_@β-CD thin films deposited at F = 300 mJ/cm^2^: surface intensity distribution of (**a**) 2930 cm^−1^ and (**b**) 1029 cm^−1^.

**Figure 7:**
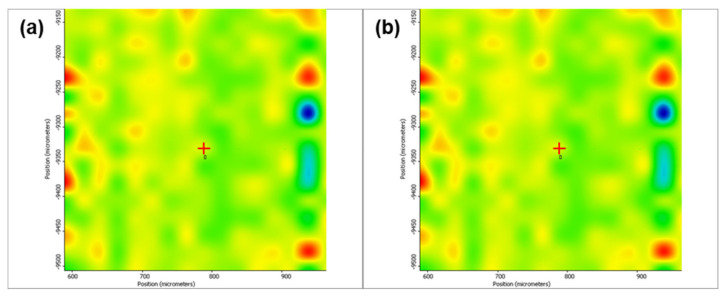
Second derivate IR mappings of Fe_3_O_4_@β-CD thin films deposited at F = 400 mJ/cm^2^: surface intensity distribution of (**a**) 2930 cm^−1^ and (**b**) 1029 cm^−1^.

**Figure 8:**
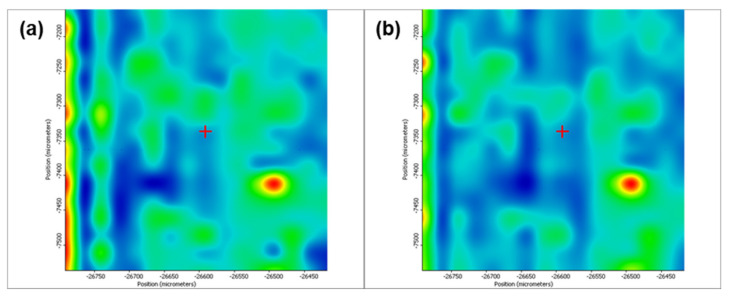
Second derivate IR mappings of Fe_3_O_4_@β-CD thin films deposited at F = 500 mJ/cm^2^: surface intensity distribution of (**a**) 2930 cm^−1^ and (**b**) 1029 cm^−1^.

**Figure 9:**
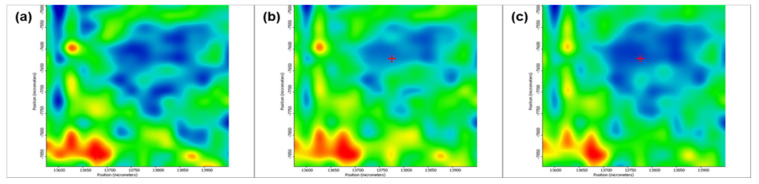
Second derivate IR mappings of Fe_3_O_4_@β-CD/PTX drop-cast: surface intensity distribution of (**a**) 2930 cm^−1^, (**b**) 1029 cm^−1^ and (**c**) ester/ketonic carbonyl vibration at 1739 cm^−1^.

**Figure 10:**
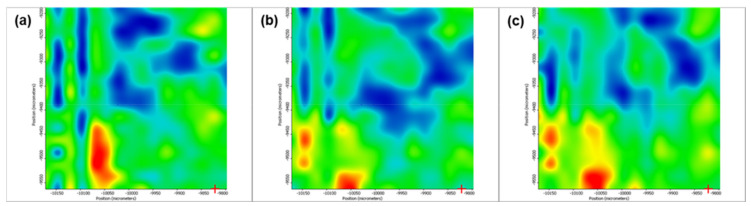
Second derivate IR mappings of Fe_3_O_4_@β-CD/PTX thin films deposited at F = 300 mJ/cm^2^: surface intensity distribution of (**a**) 2930 cm^−1^, (**b**) 1029 cm^−1^ and (**c**) ester/ketonic carbonyl vibration at 1739 cm^−1^.

**Figure 11:**
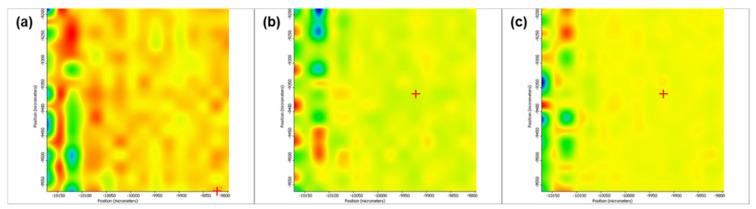
Second derivate IR mappings of Fe_3_O_4_@β-CD/PTX thin films deposited at F = 400 mJ/cm^2^: surface intensity distribution of (**a**) 2930 cm^−1^, (**b**) 1029 cm^−1^ and (**c**) 1739 cm^−1^.

**Figure 12:**
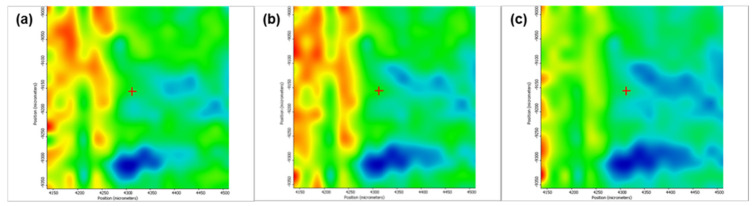
Second derivate IR mappings of Fe_3_O_4_@β-CD/PTX thin films deposited at F = 500 mJ/cm^2^: surface intensity distribution of (**a**) 2930 cm^−1^, (**b**) 1029 cm^−1^ and (**c**) 1739 cm^−1^.

Figure 5 depicts the second derivative infrared micrographs (IRM) of Fe_3_O_4_@β-CD drop-cast, while Figure 6, Figure 7 and Figure 8 plot the IRM of the same nanocomposite deposited by MAPLE tehnique at 300, 400, and 500 mJ/cm^2^, respectively. The lower intensities associated with significant chemical degradation of the monitored functional group are colored in blue, while higher intensities indicating a minimal chemical degradation are colored in red. Similar IR maps of PTX-loaded Fe_3_O_4_@β-CD composites drop-cast and MAPLE-are plotted in Figure 9, Figure 10, Figure 11 and Figure 12, respectively.

**Figure 13:**
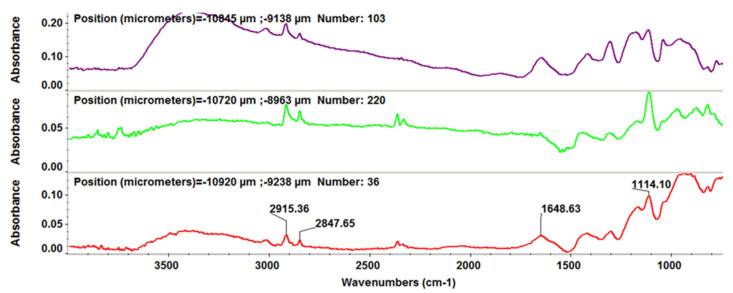
IR spectra recorded for bare Fe_3_O_4_@β-CD thin films deposited at F = 400 mJ/cm^2^.

**Figure 14:**
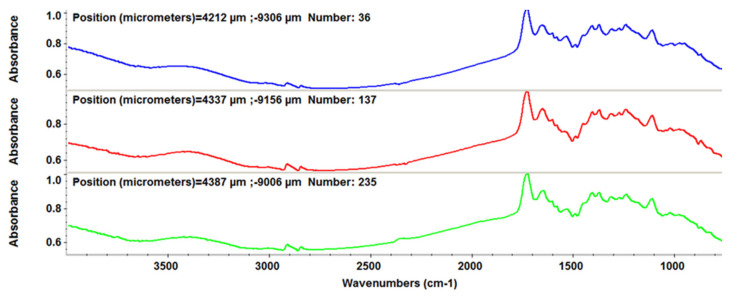
IR spectra recorded for the thin films of Fe_3_O_4_@β-CD/PTX deposited at F = 400 mJ/cm^2^.

As can be seen from the comparative analysis of IRM micrographs (Figure 5, Figure 6, Figure 7, Figure 8, Figure 9, Figure 10, Figure 11 and Figure 12), the best compromise between the deposition rate and the deposited material’s structural integrity was obtained for a laser beam fluence (F) of 400 mJ/cm^2^. IR spectra collected at different points in the surface distribution maps of spectral marker absorbance are shown for pristine (Figure 13) and Fe_3_O_4_@β-CD/PTX (Figure 14) thin films deposited at this optimal fluence value. Analysis of the FT-IR spectral data confirms the successful formation of the inclusion complex.

We analyzed these spectra comparatively, searching for differences due to PTX-specific absorptions. We focused our attention on two frequencies, namely the phenyl ring absorption band at 1475 cm^−1^ [45] and the carbonyl group absorption at 1739 cm^−1^. The PTX band at 1475 cm^−1^ is almost completely masked in the Fe_3_O_4_@β-CD/PTX nanocomposites, indicating molecular docking of the phenyl ring structural moieties into the inner hydrophobic cavity of β-CD. On the other hand, as compared to bare β-CD-modified magnetite, in the IR spectrum of PTX-loaded Fe_3_O_4_@β-CD, there appears a sharp peak at ~1740 cm^−1^ which clearly corresponds to the carbonyl moieties of PTX. Unlike the hydrophobic benzene rings, the polar carbonyl groups are most probably located outside of the inner cavity of β-CD and therefore remain unmasked. Hydrogen bonding with the outer surface OH groups slightly alters the aspect of the vibrational band of the latter. These significant spectral results were expected, being ascribed to host (β-CD)–guest (PTX) interactions in the inclusion complex.

**Figure 15:**
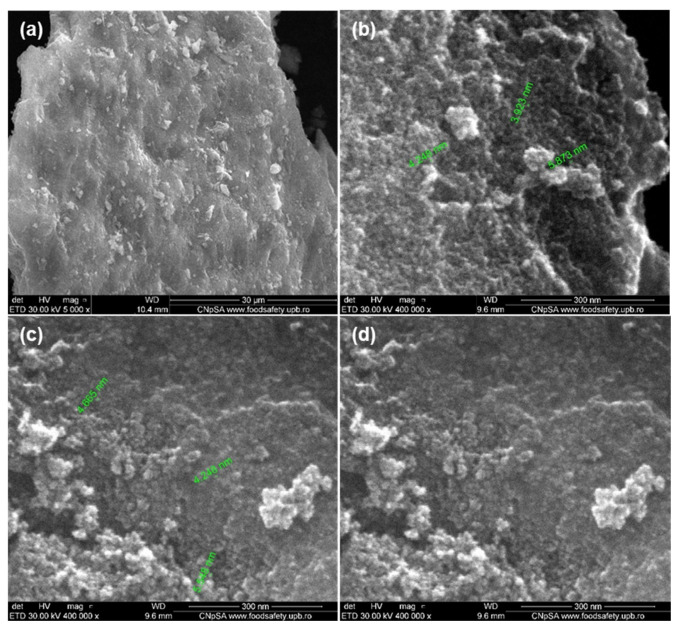
SEM micrographs of Fe_3_O_4_@β-CD nanopowders at (**a**) low-magnification (5000×) and (**b**–**d**) high-magnification (400,000×).

Surface morphological details (size and shape) of Fe_3_O_4_@β-CD nanocarrier powders were investigated by SEM. Agglomerated nanoparticles can be observed in the low-magnification (5000×) SEM micrograph (Figure 15a). High-magnification images (400,000×) revealed 3–5 nm-sized nanoparticles of well-defined spherical morphology (Figure 15b,c).

**Figure 16:**
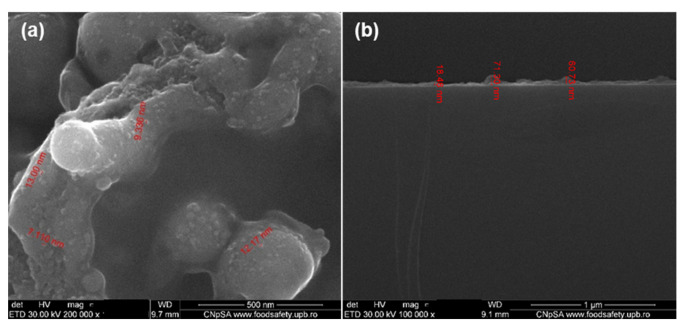
Plain view (**a**) and cross-section (**b**) SEM micrographs of Fe_3_O_4_@β-CD/PTX deposited at 400 mJ/cm^2^.

SEM analysis was also used to determine the shape, morphological and textural features of the thin films. Figure 16b show (at 100,000× magnification) the cross-section of Fe_3_O_4_@β-CD thin films deposited at 400 mJ/cm^2^ laser fluence. One can observe the uniform, compact, and agglomerated morphology of the film. The film’s surface exhibits an irregular aspect with thicknesses varying from 18 nm up to 71 nm (Figure 16b).

### 3.3. In Vitro Biocompatibility of Fe_3_O_4_@β-CD Nanocomposite Thin Films and Anti-Tumoral Activity of Paclitaxel-Loaded Films

**Figure 17:**
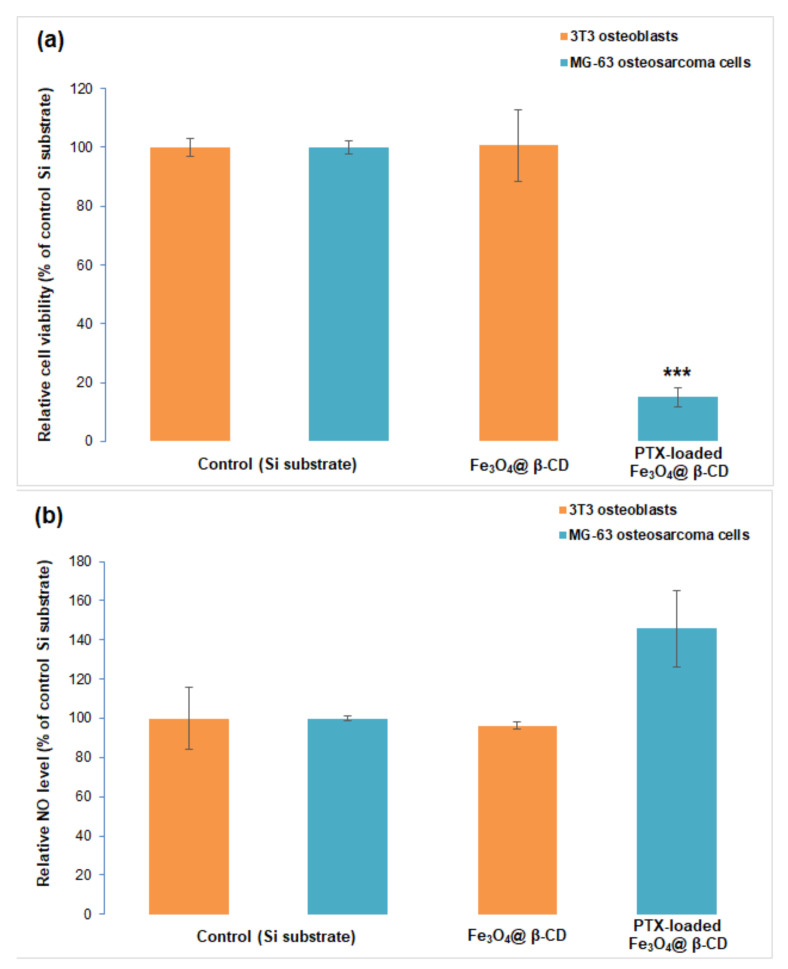
Biocompatibility of Fe_3_O_4_@β-CD nanoparticles on normal 3T3 osteoblasts (**a**) and anti-tumor efficiency of PTX-loaded Fe_3_O_4_@β-CD nanocomposites on MG-63 osteosarcoma cells (**b**) evidenced by the results of MTT (**a**) and Griess (**b**) assays. The results were calculated as mean values (*n* = 3) and expressed relative to control samples (***p < 0.001).

**Figure 18:**
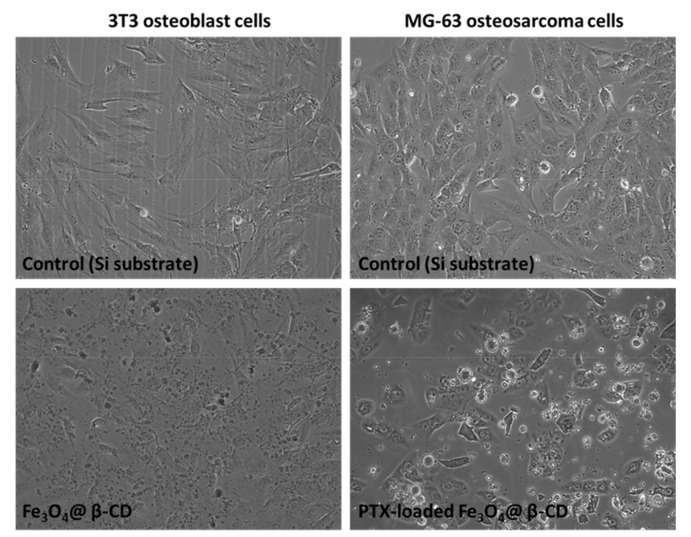
Images of phase-contrast microscopy of normal 3T3 osteoblasts and MG-63 osteosarcoma cells cultured on the surface of Fe_3_O_4_@β-CD and PTX-loaded Fe_3_O_4_@β-CD thin films, respectively. Objective used: 10×.

In order to evaluate the biocompatibility of our novel MAPLE-fabricated magnetic films represented by Fe_3_O_4_@β-CD nanoparticles, we carried out simple cytotoxicity assays on a normal 3T3 osteoblast cell line culture. No change in cell viability was observed in the presence of the bare β-CD modified magnetite after performing MTT test (Figure 17a), and the level of NO in the culture medium was similar to the control (Figure 17b). The good biocompatibility of this Fe_3_O_4_@β-CD nanocoating was also confirmed by phase-contrast microscopy, as no evident adverse effect on the morphology of normal osteoblasts was seen in Figure 18.

To evaluate the anti-tumor outcome of PTX-loaded Fe_3_O_4_@β-CD nanocomposites, the MG-63 osteosarcoma cell line was used. A significant decrease to 85% of control (represented by a simple glass slide) proved the high efficiency of PTX-loaded β-CD surface-modified SPIONs deposited as thin films by MAPLE technique on glass substrates (Figure 17a). According to a previous report, PTX induced a decrease of MG-63 osteosarcoma cell viability of only 10% over control after 24 h of incubation [35]. Comparing the poor anti-tumoral activity of the free drug with the high efficiency of PTX-loaded β-CD surface-modified SPIONs deposited as thin films by MAPLE, we concluded that these modified surface have improved activity and great potential for anti-cancer applications. The NO level rose to 145% of the control (Figure 17b), suggesting the inflammatory effect induced by these PTX-loaded nanocomposites. Only a few tumor osteoblasts were noticed after 24 h of incubation with PTX-loaded Fe_3_O_4_@β-CD nanocomposites in the phase-contrast microscopy images (Figure 18).

Regarding the design of new pharmacologically active composites for cancer therapy, it has previously been shown that PTX/hydroxypropyl-β-CD complex-loaded liposomes have the potential as drug nanocarriers to treat lung adenocarcinoma [46]. The cyclodextrin-Fe_3_O_4_ formulation was also used to encapsulate camptothecin in order to improve its solubility and bioavailability for cancer cells. It has been confirmed that this could be used as a major nanocarrier for camptothecin to effectively treat colon cancer [47].

### 3.4. In Vitro Biocompatibility of Fe_3_O_4_@β-CD Nanocomposite Thin Films on SBF

**Figure 19:**
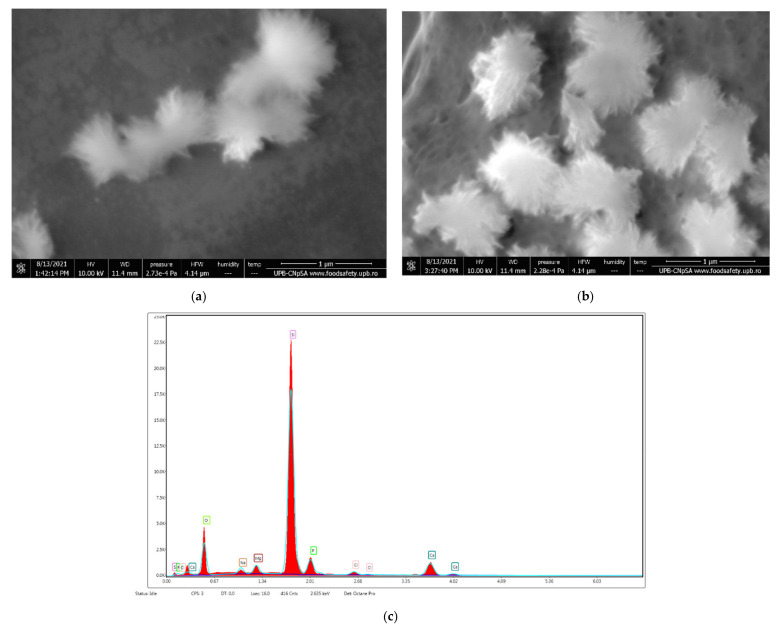
SEM images (**a**,**b**) and EDS analysis (**c**) of Fe_3_O_4_@β-CD after 14 days of incubation in SBF at 37 °C.

A supplementary in vitro test was performed on the SBF. The samples were immersed in SBF for 14 days, and subsequently, the surface of the sample was evaluated using a VERSA 3D scanning electron microscope. The main results are plotted in Figure 19. The formation of biological apatite with needle-like structures on the surface of the Fe_3_O_4_@β-CD thin coating can be seen in Figure 19a,b, and was also confirmed by EDS (Figure 19c).

## 4. Conclusions

We succeeded in the preparation of both pristine and PTX-loaded β-CD surface-modified SPIONs, not only in the form of nanopowders but also as thin films deposited by MAPLE technique. The physicochemical characterization of nanopowders was carried out using XRD, SEM, TEM, SAED, FT-IR, TGA, and DSC. The structural integrity of the thin films, deposited at the optimal laser beam fluence of 400 mJ/cm^2^, was proved by mapping the surface distribution of the FT-IR absorbances of characteristic spectral markers. Biocompatibility and cytotoxicity of the prepared magnetic hybrid nanocomposites were assessed in vitro by performing an MTT test on normal 3T3 cell line osteoblasts and osteosarcoma MG-63 cell lines. The pristine β-CD surface-modified nanopowders and thin films showed excellent biocompatibility and nocytotoxic effect. On the other hand, magnetic thin films loaded with the anti-cancer drug PTX exhibited significantly increased affinity for tumoral cells. The above properties recommend our novel magnetic nanopowders and thin films as valuable candidates for future magnetically targeted drug delivery, theragnostic, and hyperthermia applications in cancer treatment.

## Data Availability

Available from the corresponding author upon request.

## References

[1] Alifu N., Sun Z., Zebibula A., Zhu Z., Zhao X., Wu C., Wang Y., Qian J. (2017). Deep-red polymer dots with bright two-photon fluorescence and high biocompatibility for in vivo mouse brain imaging. Opt. Commun..

[2] Ferlay J., Colombet M., Soerjomataram I., Dyba T., Randi G., Bettio M., Gavin A., Visser O., Bray F. (2018). Cancer incidence and mortality patterns in Europe: Estimates for 40 countries and 25 major cancers in 2018. Eur. J. Cancer.

[3] Paul Catalin B., Alexandru Mihai G. (2015). Smart Synthetic Polymer Nanocarriers for Controlled and Site-Specific Drug Delivery. Curr. Top. Med. Chem..

[4] Paul Catalin B., Dragos G., Iulia Alexandra G. (2018). Smart Triggered Release in Controlled Drug Delivery. Curr. Drug Targets.

[5] Arzani H., Adabi M., Mosafer J., Dorkoosh F., Khosravani M., Maleki H., Nekounam H., Kamali M. (2019). Preparation of curcumin-loaded PLGA nanoparticles and investigation of its cytotoxicity effects on human glioblastoma U87MG cells. Biointerface Res. Appl. Chem..

[6] Balaure P., Gudovan D., Gudovan I., Boukherroub R., Szunerits S., Drider D. (2017). Organic polymeric nanomaterials as advanced tools in the fight against antibiotic-resistant infections. Functionalized Nanomaterials for the Management of Microbial Infection: A Strategy to Address Microbial Drug Resistance.

[7] Dragoș G., Paul Cătălin B., Dan Eduard M., Adrian F., Bogdan P., Mihai R. (2015). Functionalized magnetic nanoparticles for biomedical applications. Curr. Pharm. Des..

[8] Al Faraj A., Shaik A.P., Shaik A.S. (2015). Effect of surface coating on the biocompatibility and in vivo MRI detection of iron oxide nanoparticles after intrapulmonary administration. Nanotoxicology.

[9] Dulińska-Litewka J., Łazarczyk A., Hałubiec P., Szafrański O., Karnas K., Karewicz A. (2019). Superparamagnetic Iron Oxide Nanoparticles—Current and Prospective Medical Applications. Materials.

[10] Liong M., Lu J., Kovochich M., Xia T., Ruehm S.G., Nel A.E., Tamanoi F., Zink J.I. (2008). Multifunctional Inorganic Nanoparticles for Imaging, Targeting, and Drug Delivery. ACS Nano.

[11] Jain T.K., Richey J., Strand M., Leslie-Pelecky D.L., Flask C.A., Labhasetwar V. (2008). Magnetic nanoparticles with dual functional properties: Drug delivery and magnetic resonance imaging. Biomaterials.

[12] Alghuthaymi M. (2020). Magnetic-silica nanoshell for extraction of fungal genomic DNA from *Rhizopus oryzae*. Biointerface Res. Appl. Chem..

[13] Mohamad A., Rizwan M., Keasberry N.A., Ahmed M.U. (2019). Fabrication of label-free electrochemical food biosensor for the sensitive detection of ovalbumin on nanocomposite-modified graphene electrode. Biointerface Res. Appl. Chem..

[14] Wang Y.X. (2011). Superparamagnetic iron oxide based MRI contrast agents: Current status of clinical application. Quant. Imaging Med. Surg..

[15] Silva A.C., Oliveira T.R., Mamani J.B., Malheiros S.M.F., Malavolta L., Pavon L.F., Sibov T.T., Amaro Junior E., Tannus A., Vidoto E.L.G. (2011). Application of hyperthermia induced by superparamagnetic iron oxide nanoparticles in glioma treatment. Int. J. Nanomed..

[16] Laurent S., Forge D., Port M., Roch A., Robic C., Vander Elst L., Muller R.N. (2008). Magnetic iron oxide nanoparticles: Synthesis, stabilization, vectorization, physicochemical characterizations, and biological applications. Chem. Rev..

[17] Elazab H.A., Gadalla M.A., Sadek M.A., El-Idreesy T.T. (2019). Hydrothermal synthesis of graphene supported Pd/Fe3O4 nanoparticles as efficient magnetic catalysts for Suzuki Cross—Coupling. Biointerface Res. Appl. Chem..

[18] Rajendrachari S., Ceylan K.B. (2020). The activation energy and antibacterial investigation of spherical Fe3O4 nanoparticles prepared by Crocus sativus (Saffron) flowers. Biointerface Res. Appl. Chem..

[19] Haroun A.A., Ahmed E.F., Hakeim O.A. (2020). Multifunctional hyperbranched polyester grafted beta-cyclodextrin metal complexes for textile coating. Biointerface Res. Appl. Chem..

[20] Haiahem S., Abdelaziz B., Leila N., Imene D., Madi F., Eddine K. (2012). Molecular docking study on β-cyclodextrin Interactions of metobromuron and [3-(p-bromophenyl)-1-methoxy-1-methylurea]. J. Incl. Phenom. Macrocycl. Chem..

[21] Chen P., Yao S., Chen X., Huang Y., Song H. (2019). A new strategy for the construction of β-cyclodextrin-based magnetic nanocarriers: A molecular docking technique. New J. Chem..

[22] Jayaprabha K.N., Joy P.A. (2015). Citrate modified β-cyclodextrin functionalized magnetite nanoparticles: A biocompatible platform for hydrophobic drug delivery. RSC Adv..

[23] Jeon H., Kim J., Lee Y.M., Kim J., Choi H.W., Lee J., Park H., Kang Y., Kim I.S., Lee B.H. (2016). Poly-paclitaxel/cyclodextrin-SPION nano-assembly for magnetically guided drug delivery system. J. Control. Release Off. J. Control. Release Soc..

[24] Monteiro A.P.F., Caminhas L.D., Ardisson J.D., Paniago R., Cortés M.E., Sinisterra R.D. (2017). Magnetic nanoparticles coated with cyclodextrins and citrate for irinotecan delivery. Carbohydr. Polym..

[25] Chen P., Song H., Yao S., Tu X., Su M., Zhou L. (2017). Magnetic targeted nanoparticles based on β-cyclodextrin and chitosan for hydrophobic drug delivery and a study of their mechanism. RSC Adv..

[26] Bai L., Zhao Q., Wang J., Gao Y., Sha Z., Di D., Han N., Wang Y., Zhang J., Wang S. (2015). Mechanism study on pH-responsive cyclodextrin capped mesoporous silica: Effect of different stalk densities and the type of cyclodextrin. Nanotechnology.

[27] Mrówczyński R., Jędrzak A., Szutkowski K., Grześkowiak B.F., Coy E. (2018). Cyclodextrin-Based Magnetic Nanoparticles for Cancer Therapy. Nanomaterials.

[28] Shen L., Li B., Qiao Y. (2018). Fe₃O₄ Nanoparticles in Targeted Drug/Gene Delivery Systems. Materials.

[29] Piehler S., Dähring H., Grandke J., Göring J., Couleaud P., Aires A., Cortajarena A.L., Courty J., Latorre A., Somoza Á. (2020). Iron Oxide Nanoparticles as Carriers for DOX and Magnetic Hyperthermia after Intratumoral Application into Breast Cancer in Mice: Impact and Future Perspectives. Nanomaterials.

[30] Vangijzegem T., Stanicki D., Laurent S. (2019). Magnetic iron oxide nanoparticles for drug delivery: Applications and characteristics. Expert Opin. Drug Deliv..

[31] Badruddoza A.Z.M., Rahman M.T., Ghosh S., Hossain M.Z., Shi J., Hidajat K., Uddin M.S. (2013). β-Cyclodextrin conjugated magnetic, fluorescent silica core-shell nanoparticles for biomedical applications. Carbohydr. Polym..

[32] Hu Q.-D., Tang G.-P., Chu P.K. (2014). Cyclodextrin-Based Host–Guest Supramolecular Nanoparticles for Delivery: From Design to Applications. Acc. Chem. Res..

[33] Grumezescu V., Socol G., Grumezescu A.M., Holban A.M., Ficai A., Trusca R., Bleotu C., Balaure P.C., Cristescu R., Chifiriuc M.C. (2014). Functionalized antibiofilm thin coatings based on PLA-PVA microspheres loaded with usnic acid natural compounds fabricated by MAPLE. Appl. Surf. Sci..

[34] Pereira C., Pereira A.M., Fernandes C., Rocha M., Mendes R., Fernández-García M.P., Guedes A., Tavares P.B., Grenèche J.-M., Araújo J.P. (2012). Superparamagnetic MFe_2_O_4_ (M = Fe, Co, Mn) Nanoparticles: Tuning the Particle Size and Magnetic Properties through a Novel One-Step Coprecipitation Route. Chem. Mater..

[35] Liu S.Y., Song S.X., Lin L., Liu X. (2010). Molecular Mechanism of Cell Apoptosis by Paclitaxel and Pirarubicin in a Human Osteosarcoma Cell Line. Chemotherapy.

[36] Piqué A., McGill R.A., Chrisey D., Leonhardt D., Mslna T.E., Spargo B., Callahan J., Vachet R.W., Chung R., Bucaro M. (1999). Growth of organic thin films by the matrix assisted pulsed laser evaporation (MAPLE) technique. Thin Solid Film..

[37] Kokubo T., Takadama H. (2006). How useful is SBF in predicting in vivo bone bioactivity?. Biomaterials.

[38] Zhai Y., Liu F., Zhang Q., Gao G. (2009). Synthesis of magnetite nanoparticle aqueous dispersions in an ionic liquid containing acrylic acid anion. Colloids Surf. A Physicochem. Eng. Asp..

[39] Kemelbekov U., Luo Y., Orynbekova Z., Rustembekov Z., Haag R., Saenger W., Praliyev K. (2011). IR, UV and NMR studies of β-cyclodextrin inclusion complexes of kazcaine and prosidol bases. J. Incl. Phenom. Macrocycl. Chem..

[40] Chen J., Qin X., Zhong S., Chen S., Su W., Liu Y. (2018). Characterization of Curcumin/Cyclodextrin Polymer Inclusion Complex and Investigation on Its Antioxidant and Antiproliferative Activities. Molecules.

[41] Paczkowska M., Mizera M., Piotrowska H., Szymanowska-Powałowska D., Lewandowska K., Goscianska J., Pietrzak R., Bednarski W., Majka Z., Cielecka-Piontek J. (2015). Complex of Rutin with β-Cyclodextrin as Potential Delivery System. PLoS ONE.

[42] Stoia M., Istratie R., Păcurariu C. (2016). Investigation of magnetite nanoparticles stability in air by thermal analysis and FTIR spectroscopy. J. Therm. Anal. Calorim..

[43] Velázquez N.S., Ferreyra M.G., Mengatto L.N., Santagapita P.R., Buera M.P., Luna J.A. (2019). Paclitaxel/β-Cyclodextrin interactions, a perspective from pulsed NMR spectroscopy experiments. Carbohydr. Res..

[44] Loh G.O.K., Tan Y.T.F., Peh K.-K. (2016). Enhancement of norfloxacin solubility via inclusion complexation with β-cyclodextrin and its derivative hydroxypropyl-β-cyclodextrin. Asian J. Pharm. Sci..

[45] Ye Y.J., Wang Y., Lou K.Y., Chen Y.Z., Chen R., Gao F. (2015). The preparation, characterization, and pharmacokinetic studies of chitosan nanoparticles loaded with paclitaxel/dimethyl-β-cyclodextrin inclusion complexes. Int. J. Nanomed..

[46] Shen Q., Shen Y., Jin F., Du Y.Z., Ying X.Y. (2020). Paclitaxel/hydroxypropyl-β-cyclodextrin complex-loaded liposomes for overcoming multidrug resistance in cancer chemotherapy. J. Liposome Res..

[47] Krishnan P., Rajan M., Kumari S., Sakinah S., Priya S.P., Amira F., Danjuma L., Pooi Ling M., Fakurazi S., Arulselvan P. (2017). Efficiency of newly formulated camptothecin with β-cyclodextrin-EDTA-Fe3O4 nanoparticle-conjugated nanocarriers as an anti-colon cancer (HT29) drug. Sci. Rep..

